# Effects of Intravenous Bolus Injection of Fentanyl on Phrenic Nerve Activity and Its Response to Hypoxia and Hypercapnia

**Published:** 2026-04-04

**Authors:** Jianguo Zhuang, Xiuping Gao, Shan Shi, Fadi Xu

**Affiliations:** Department of Physiology, Lovelace Biomedical Research Institute, Albuquerque, NM 87108

**Keywords:** Inspiratory motor drive, vagotomy, cardiovascular activity

## Abstract

Intravenous bolus (IVb) injection of overdose fentanyl triggers an immediate apnea followed by severely depressed ventilation with several associated effects, such as upper airway constriction, respiratory muscle rigidity, hypoxemia/hypercapnia (due to hypoventilation) and hypothermia. This study sought to determine the direct impact of IVb injection of fentanyl on phrenic nerve activity (PN) over time in anesthetized, vagotomized, paralyzed and ventilated rats to avoid the associated effects. The integrative PN (ʃPN), frequency of ʃPN (f_R_), minute PN (MPN, ∫PN × f_R_), heart rate (HR), arterial blood pressure (ABP), SpO_2_ and P_ET_CO_2_ and their responses to hypoxia (10% O_2_ in nitrogen for 1 min) and then hypercapnia (10% CO_2_ in 30% O_2_ and 60% nitrogen for 3 min) were recorded before and after IVb injection of fentanyl (30 μl/kg). Fentanyl induced an extra long-lasting apnea (for 6 min) with an-18 sec latency followed by sustained and stable MPN depression (↓70%), hypertension and tachycardia. Hypoxia (decreasing SpO_2_ by 30%) augmented MPN associated with slight but significant hypotension and tachycardia. The PN responses were strikingly reduced and the cardiovascular responses exacerbated by fentanyl. Hypercapnia (increasing P_ET_CO_2_ to 70 torr) also enhanced MPN associated with hypertension and bradycardia, and the MPN responses were blunted with the evoked hypertension turned to hypotension and the bradycardia aggravated after fentanyl. We conclude that fentanyl tremendously reduces the inspiratory motor drive partially via attenuating the chemoreflexes, which is responsible for the ventilatory depression and failure observed in the clinical setting.

## Summary Statement

Rapid intravenous injection of overdose fentanyl triggers an immediate apnea, leading to sudden death. In this study, we demonstrate that fentanyl greatly reduces the inspiratory motor drive partially via attenuating the chemoreflexes, which is likely responsible for the ventilatory depression and failure observed in the clinical setting.

## Introduction

Fentanyl is the most frequently used, highly potent and selective mu-opioid receptor (MOR) agonist capable of crossing the blood-brain-barrier. Rapid intravenous (IV) injection of overdose fentanyl can lead to sudden death within a few minutes, especially in illicit users, and constitute the majority of overdose opioid-induced deaths (https://www.cdc.gov/nchs/nvss/vsrr/drug-overdose-data.htm) [1–3]; however, the relevant mechanisms are not fully understood. We have recently established a rat model of sudden death induced by IV bolus (IVb) injection of fentanyl. Fentanyl via acting on MORs triggers an immediate apnea followed by subsequent ventilatory depression in spontaneously breathing rats [4–6], resulting in sudden death if a lethal dose is applied [7]. It is well documented that fentanyl also has other associated effects that can secondarily affect ventilation. First, fentanyl produces respiratory muscle rigidity and upper airway constriction [4, 8] that limit the ventilation. Second, opioid-induced ventilatory depression (apnea) results in hypoxemia and hypercapnia [9, 10]. Third, fentanyl brings about hypothermia [11–13]. Importantly, brain hypothermia [14–16] and hypoxemia/hypercapnia [17–20] are able to advance cardiorespiratory failure and arrest. Although MOR agonists (such as fentanyl) are known to suppress pontomedullary inspiratory neural discharges and the phrenic nerve activity (PN) [21–23], the impact of IVb injection of fentanyl on the PN over time without the associated effects as mentioned above is unknown.

Blood gas homeostasis is maintained by sufficient hypoxic (HVR) and hypercapnic ventilatory response (HCVR). Studies have demonstrated that opioids are capable of depressing HVR and HCVR via activation of MORs in humans [24–26] and mammalian species, including dogs [27], cats [28], rabbits [29], rats [30–33] and mice [34]. Furthermore, these depressed chemoreflexes are thought to substantially contribute to opioid-induced ventilatory depression [30, 35]. To date, the impact of IVb injection of fentanyl on the PN response to hypoxia and hypercapnia has not yet been investigated.

To address the above issues, the baseline PN and its responses to hypoxia and hypercapnia were recorded in the anesthetized, vagotomized, paralyzed and artificially ventilated rats before and after IVb injection of fentanyl. This animal preparation was used to prevent the interferences of the fentanyl-induced upper airway constriction, respiratory muscles rigidity, hypoxia/hypercapnia and hypothermia.

## Methods

### Animal Use

Ten pathogen-free Sprague-Dawley adult male rats were purchased from Charles River Laboratories, Inc. (Wilmington, MA). All rats were housed in filter top cages in the animal facility of Lovelace Biomedical Research Institute with a 12:12 h light/dark cycle and provided with water and food ad libitum. The rooms were constantly ventilated and the temperature was kept at 24–25°C. The animals were quarantined for one week before experiments. Experiments were performed during 9:00 and 17:00 hours to avoid influence of the circadian rhythm. The experimental protocols (FY23–010) were conducted in accordance with the Guide for the Care and Use of Laboratory Animals and approved by the Institutional Animal Care and Use Committee (IACUC), which is accredited by the Association for Assessment and Accreditation of Laboratory Animal Care International, USA.

### General Animal Preparation

The animals were anesthetized with urethane (1.2 g/kg, ip) along with supplemental doses (0.4 g/kg, ip) administered if needed to completely suppress eye-blink and limb-withdrawal reflexes. The rats were placed in a supine position and tracheotomy was performed below the larynx by blunt dissection for tracheal cannulation [31]. The latter was connected to the outlet of the ventilator (7025 Rodent Ventilator, UGO Basile SRL, Italy). The inlet of the ventilator was connected with a pneumotachograph (Cat# MLT1L, ADInstruments Inc., Colorado Springs, CO) to record airflow and loosely jacked by a tubing (diameter 2.0 cm) that was connected to a gas mixing flow-meter (GF-3MP, Cameron Instrument Co., Port Aransas, TX) for delivery of a given gas mixture (30% O_2_ in nitrogen as baseline). A CO_2_-sensor (Nonin LifeSense^®^ Vet) was utilized to sample the air through a branch of the tracheal cannula to measure partial pressure of end-tidal carbon dioxide (P_ET_CO_2_). A paw wrap sensor (Nonin Pulse Oximeter 2500V) was employed to analyze peripheral capillary oxygen saturation (SpO_2_). The right jugular vein was isolated and a catheter (PE50) was advanced close to the right atrium [6] for IVb injection. The inserted depth of the catheter was determined by measuring the distance from the heart (feel from the heartbeat) to the cannulating site. The left femoral artery was cannulated (PE-50) for recording arterial blood pressure (ABP) and heart rate (HR) via a reusable BP transducer (ADInstruments Inc. item # MLT0380/D). The animal’s core temperature (T_B_) was monitored with a rectal probe and maintained at 36.5–37.0°C by a heating pad and radiant heat lamp throughout the experiment.

### Recording of phrenic nerve activities

Animals were paralyzed with pancuronium (0.1–0.3 mg/kg for induction and 0.1 mg/kg/h for maintenance) and artificially ventilated following transection of bilateral vagus nerves. The artificial ventilation (V_T_, ~10 ml/kg at a rate of 60 −70 breaths/min) was adjusted to maintain P_ET_CO_2_ at approximately 40 torr. A positive end-expiratory pressure of 1.0 cmH_2_O was maintained to prevent atelectasis. The gas mixture (30%O_2,_ hypoxic or hypercapnic gas mixtures) flowing into the inlet of the ventilator was controlled by selecting the preset program in the mixing flow-meter. The vagotomy was conducted to prevent entrainment of phrenic motor output with the ventilator. During paralysis, the absence of nociception was evaluated and adjusted by ensuring a lack of respiratory, ABP and HR responses to firmly pinching the hind toes. The phrenic nerve on the left side was isolated, cut distally and desheathed. Its central end was mounted on a bipolar recording electrode and then covered with petroleum jelly to prevent drying. Raw signals of PN were band-pass filtered (100 – 3k Hz), amplified and recorded. Integrated PN (ʃPN) was simultaneously calculated from the raw signal integration using a 0.1 s time decay. All of the recording settings were kept the same throughout the experiment in the individual rats. ʃPN (i.e., the height), frequency of ʃPN (f_R_) and minute PN (MPN = ∫PN × f_R_), somewhat equivalent to tidal volume (V_T_), respiratory frequency (f_R_) and minute ventilation (V_E_), were measured and calculated [36]. The ∫PN and MPN were expressed in arbitrary units (a.u. and a.u./min) in the results. These signals, coupled with airflow, ABP, HR, SpO_2_, P_ET_CO_2_ and T_B_ were digitized and recorded using a PowerLab/8sp unit (model ML 785, ADInstruments Inc., Colorado Springs, CO) and a computer with LabChart Pro 7 software. An apnea is defined as the absence of PN for a period equal to or greater than three complete respiratory cycles [6].

### Chemicals

Urethane (item # U2500) were purchased from Sigma-Aldrich (St. Louis, MO). Fentanyl citrate was purchased from Sigma-Aldrich (St. Louis, MO). It was prepared in saline to a stock solution (0.4 mg mL^−1^) and subsequently diluted as needed.

### Experiment Protocols

#### Pilot Study.

It is generally accepted that the initial HVR (within 1 min of hypoxia) is mediated by the carotid body [37, 38], while 3–5 min hypercapnia-induced HCVR is mainly mediated by central CO_2_-chemoreceptors [39, 40]. Moreover, such HVR and HCVR are reproducible within the individual over time in anesthetized rats [31–33]. This pilot study sought to confirm the reproducibility of these responses in the anesthetized, vagotomized, paralyzed and ventilated rats. The cardiorespiratory activities before and during hypoxia (10% O_2_ in 90% nitrogen for 1 min) and then hypercapnia (10% CO_2_ in 30% O_2_ and 60% nitrogen for 3 min) were recorded in three rats. The two chemical challenges were apart from a-6 min interval, and the same protocols were repeated 20 min later.

#### Main Study.

This study was designed to verify the cardiorespiratory activities and their responses to hypoxia and hypercapnia before and after IVb injection of fentanyl. Cardiorespiratory activities (PN, HR, ABP, SpO_2_, P_ET_CO_2_, and T_B_) were recorded in seven rats. When the baseline activity became stable for at least 3 min, IVb injection of saline (0.1 ml, as Ctrl) was carried out. Approximately 11 min postinjection, the animal was exposed to hypoxia and then hypercapnia as mentioned in the pilot study. After twenty min, the same protocols were executed with the exception that saline was replaced by fentanyl (30 μg/kg). The time-points chosen for conduction of hypoxia and hypercapnia are based on two factors: 1) there was a stabilized ventilatory depression by IVb injection of fentanyl (up to 25 μg/kg) from 5–25 min postinjection in anesthetized rats [4] and 2) the PN responses to hypoxia and hypercapnia were full recovered within 6 min and these responses were reproducible in our pilot study.

### Data Analysis

The data showing the cardiorespiratory activities (MPN, ∫PN, f_R_, ABP, HR, SpO_2_, P_ET_CO_2_ and T_B_) and their response to hypoxia and hypercapnia before and after fentanyl were collected in two ways. First, the cardiorespiratory changes over time by fentanyl were divided into three phases: before, during, and after the extra long-lasting silent PN (apnea). Because of the considerable variations of the latency and the duration of the evoked apnea among the individuals, the times when the apnea started and ended were first defined in each animal (i.e., two time-points) to correctly reflect the apnea as previously reported [41]. Besides the “0” (as baseline), one time-point was selected 5 breaths before the apnea and four time-points were collected approximately every min after the apnea for ~4 min. Therefore, there were 8 time-points (T1 - T8) to display the fentanyl effect over time and the averaged time at each time-point (T1 – T8) and its variation were detailed in Table 1. The Ctrl data (before fentanyl) were collected at the same averaged time. In all rats, the cardiorespiratory values at each time-point were measured from 5 sec signals and were expressed as the absolute values. Second, the response to hypoxia was determined by the largest response during the earlier phase of hypoxia and to hypercapnia as defined by the greatest response during hypercapnia in each animal. These data were presented as Δchange from the corresponding baseline data. Group data were reported as means ± SE. Two-way ANOVA with repeated measures was used to analyze the differences of the baseline data between the rats receiving injection of vehicle and fentanyl over time, as well as the hypoxia- or hypercapnia-induced change (Δchange) vs. “0” (no change) in each group and between the two groups. The Benjamini-Hochberg method to control the false discovery rate, with P-values < 0.05, was considered significant

Table 1 presents the averaged time and its variation when the corresponding data are collected over time in the anesthetized, vagotomized, paralyzed and ventilated rats. The data for: time-points 1 and 2 (T1 and T2, Phase I) were collected just before fentanyl injection (as baseline) and five breaths before the fentanyl-induced apnea; T3-T4 (Phase II) were obtained at the beginning and the end of apnea; and T5-T8 (Phase III) were measured approximately every 1 min after the restoration of the respiratory rhythm just before hypoxia (FNT = fentanyl; n = 7).

## Results

### The phrenic nerve responses to hypoxia and hypercapnia are reproducible

**Figure 1:**
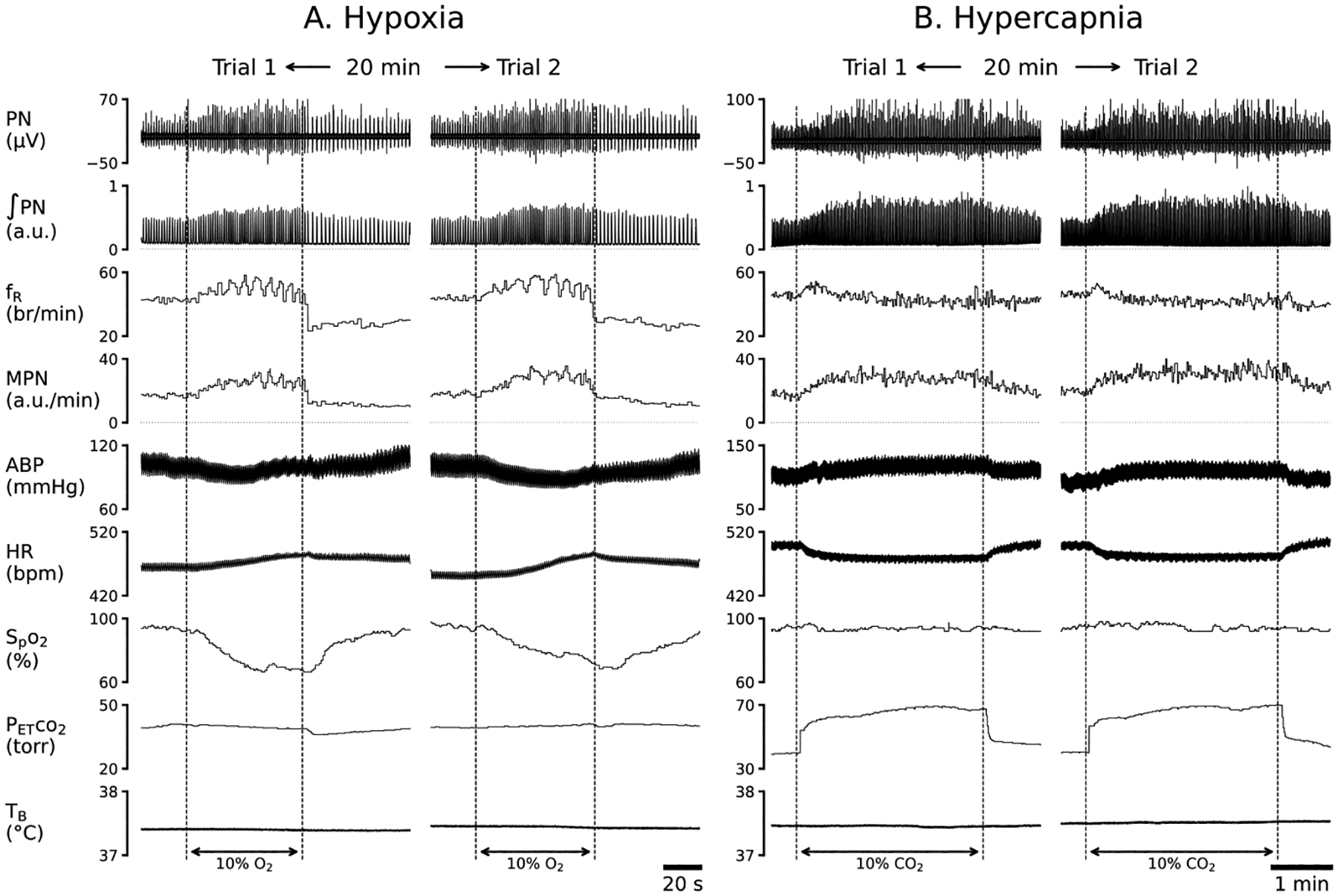
The representative recordings of cardiorespiratory responses to hypoxia (10% O_2_ for 1 min, A) and hypercapnia (10% CO_2_ for 3 min, B) in an anesthetized, vagotomized, paralyzed and ventilated rat and their repetitions 20 min later (trial 2). The traces from the top to bottom are phrenic nerve activity (PN), integrated PN (ʃPN), frequency of ʃPN (f_R_) and minute integrated PN (MPN), partial pressure of end-tidal carbon dioxide (P_ET_CO_2_), arterial blood pressure (ABP), heart rate (HR), peripheral capillary oxygen saturation (SpO_2_), and body temperature (T_B_). These parameters are the same for the following figures.

Our pilot study was conducted to confirm the reproducibility of the cardiorespiratory responses to hypoxia and hypercapnia in three anesthetized, vagotomized, paralyzed and ventilated rats. As exhibited in Fig. 1, hypoxic (10% O_2_ for 1 min) produced remarkable increases in MPN, ʃPN and f_R_ with slight hypotension and tachycardia, and hypercapnia (10% CO_2_ for 3 min) promoted similar PN response (slight f_R_ enhancement) associated with hypertension and bradycardia. These hypoxic and hypercapnic responses were fully recovered within 5 min and both chemoreflexes were reproducible. Thus, the reproducibility of the evoked PN responses within the individuals are similar to that of the ventilatory responses to hypoxia and hypercapnia in anesthetized and spontaneously breathing rats with the vagal intact [31–33].

### Fentanyl induces an extra long-lasting apnea followed by severe depression of PN

**Figure 2:**
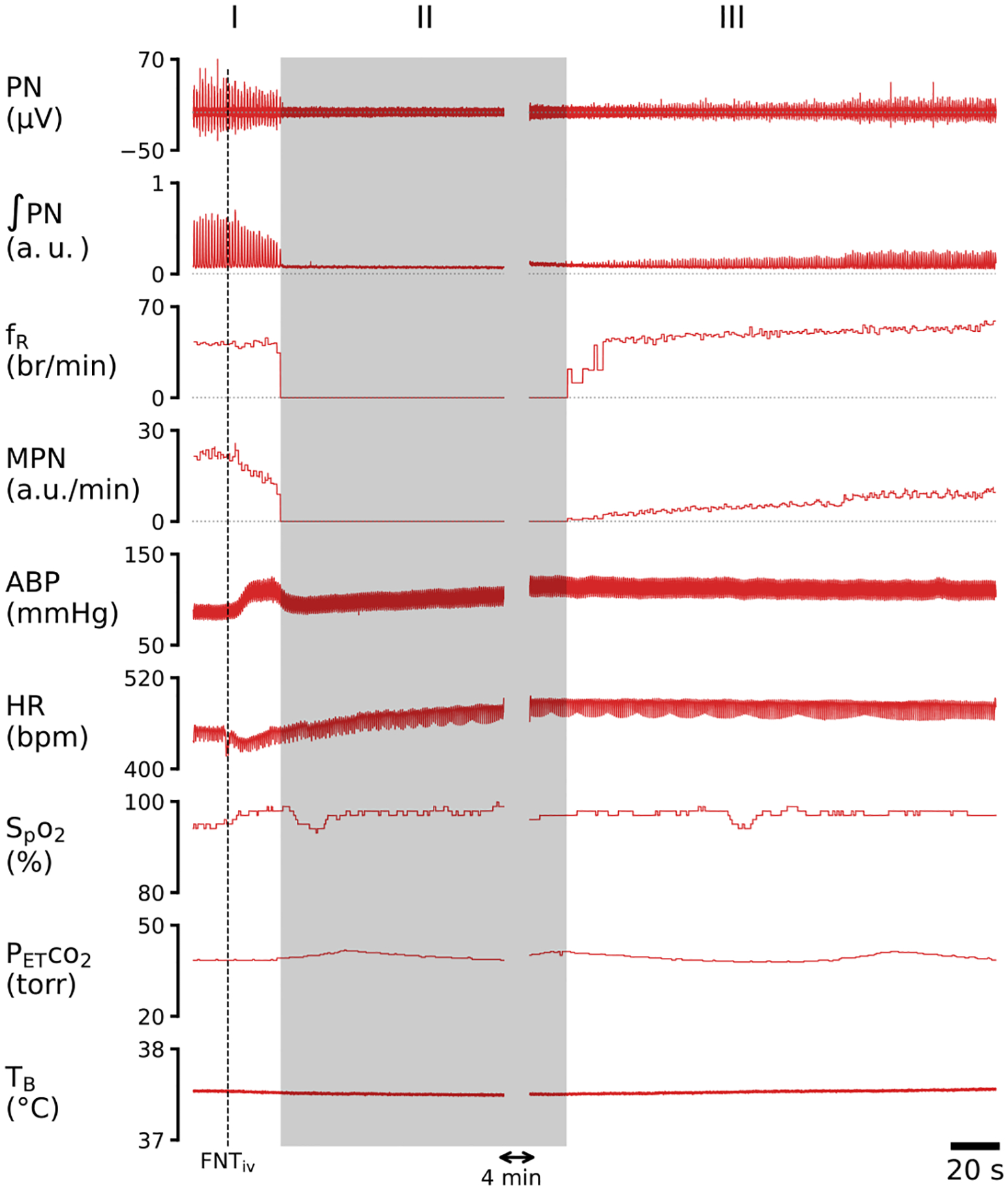
Typical recordings of cardiorespiratory activities before and after IVb injection of fentanyl (FNTiv, 30 μg/kg) in an anesthetized, vagotomized, paralyzed and ventilated rat. I, II and III represent three phases of the cardiorespiratory response.

**Figure 3:**
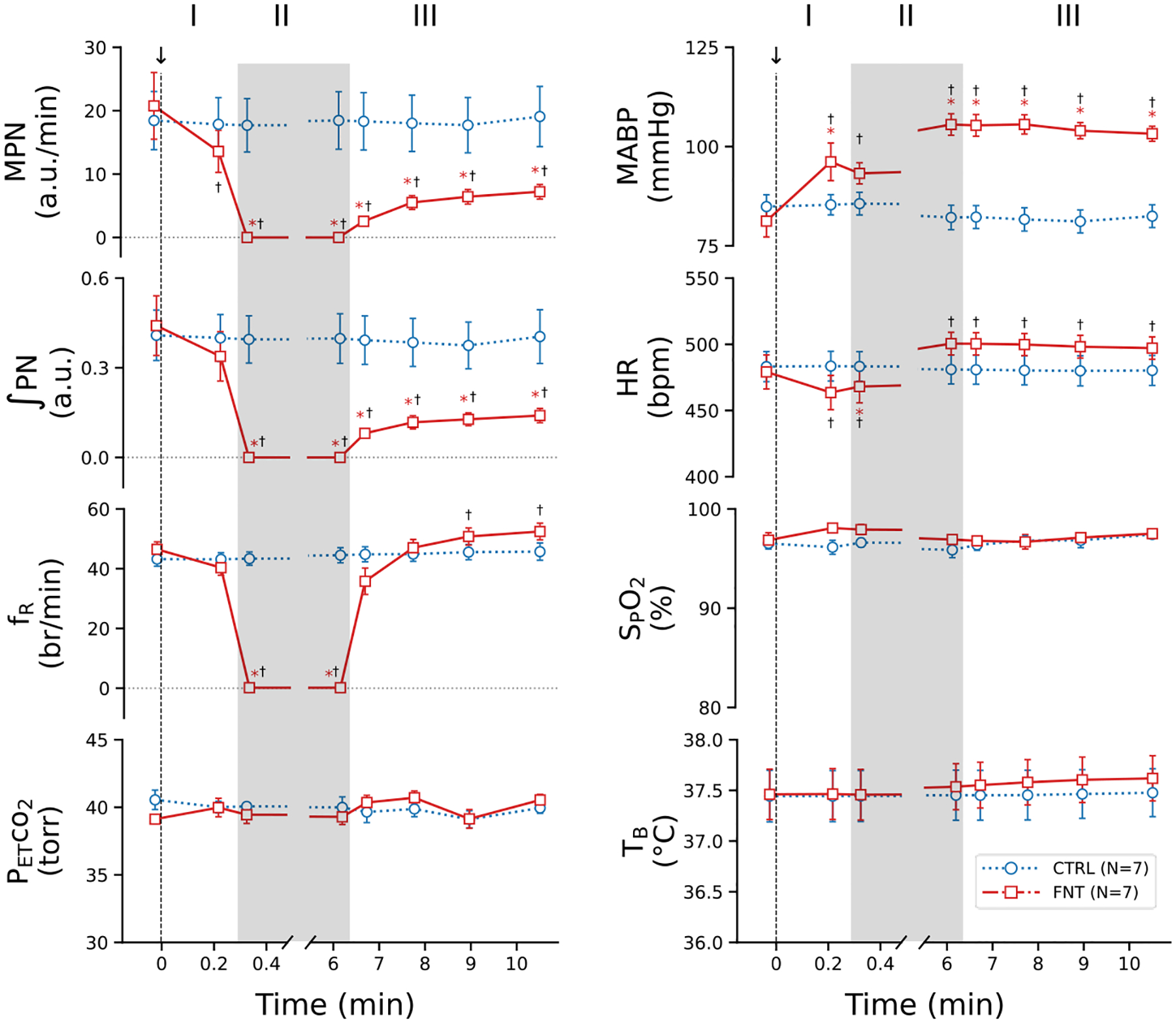
The group data of the cardiorespiratory activities before (baselines, before “0 min”) and after IVb injection of vehicle or fentanyl (CTRL or FNT). The arrows, along with the dash lines, point to intravenous bolus injection saline or FNT. I, II and III represent three phases of the cardiorespiratory response. n = 7; mean ± SE; *, P < 0.05 compared to baseline values and †, P < 0.05 compared to CTRL.

Fig. 2 presents the typical recordings of the cardiorespiratory changes induced by IVb injection of fentanyl. Fentanyl elicited robust cardiorespiratory changes, which were characterized by the following activity: 1) the initial quick decline of ʃPN, leading to an apnea, associated with hypertension and bradycardia (Phase I); 2) an extra long-lasting apnea (for 5.7 min) accompanied with further hypertension and tachycardia (Phase II); and 3) the subsequent sustained depression of MPN and ʃPN as well as hypertension and tachycardia with f_R_ recovered rapidly once the respiratory rhythm was restored (Phase III). The corresponding group data are illustrated in Fig. 3. The baseline cardiorespiratory activities (at time “0”) were not significantly different in rats before IVb injection of saline and fentanyl. Compared to the Ctrl (vehicle injection), fentanyl diminished MPN, leading to the apnea within 18 ± 0.8 sec, and the apnea lasted for 6.2 ± 0.25 min followed by a severe and stabilized depression of MPN and ʃPN (↓~70%) at the end of the experiment (~11 min postinjection). Interestingly, after restoration of the respiratory rhythm, f_R_ was fully recovered and slightly higher than the baseline values within ~1 min post-apnea. Regarding the cardiovascular change, fentanyl evoked hypertension and tachycardia after a brief bradycardia.

### IVb injection of fentanyl blunts the cardiorespiratory response to hypoxia

**Figure 4:**
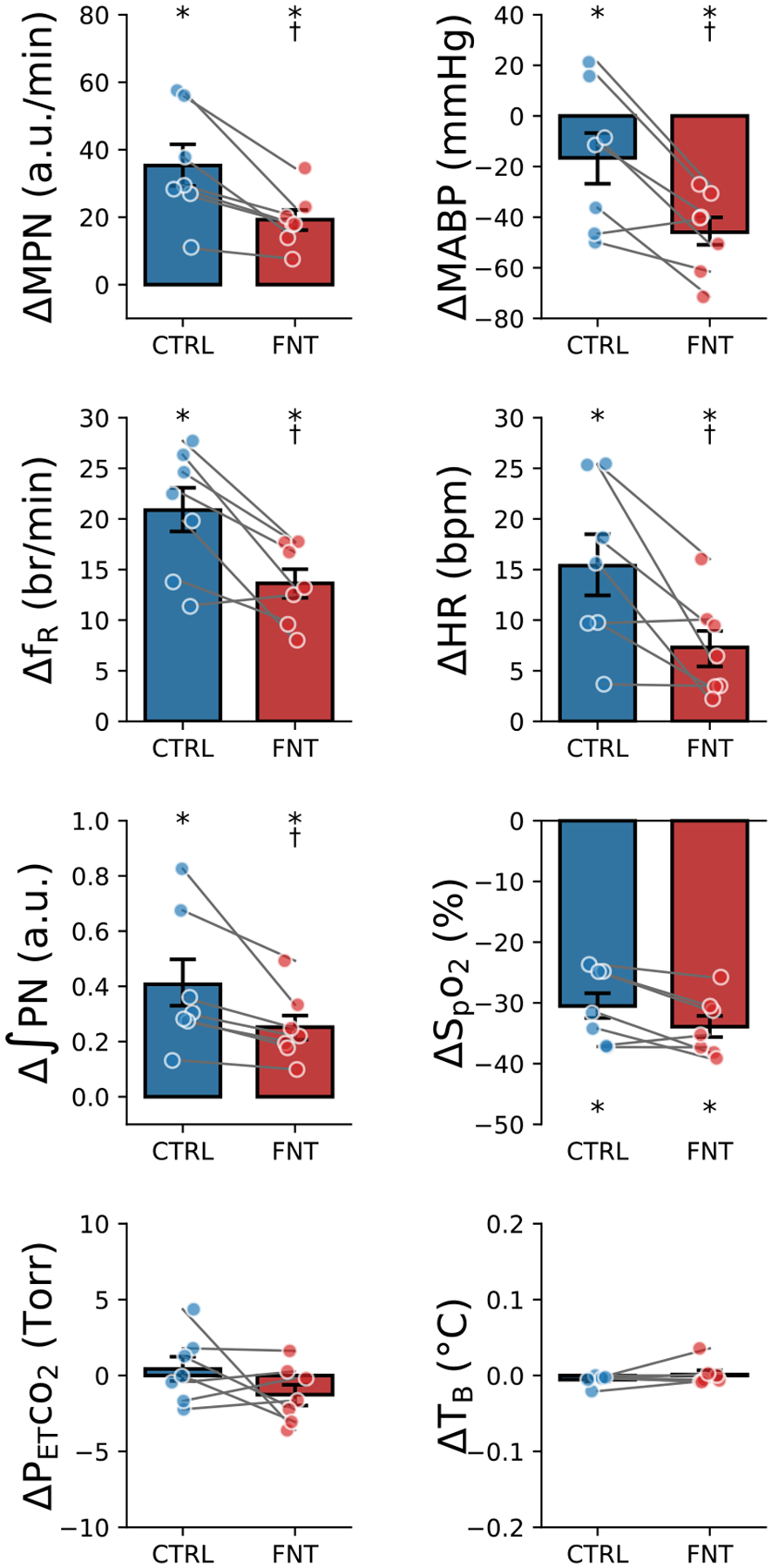
Cardiorespiratory responses to hypoxia (10% O_2_ for 1 min) before (CTRL) and after IVb injection of fentanyl (FNT) in anesthetized, vagotomized, paralyzed and ventilated rats. n = 7. mean ± SE; *, P < 0.05 compared to before hypoxia; †, P < 0.05 compared to CTRL.

As described above, the PN was not affected by injection of saline, but it was suppressed by 70% at ~11 min postinjection of fentanyl. We subsequently tested the cardiorespiratory response to hypoxia before (Ctrl) and after fentanyl and results are depicted in Fig 4. Hypoxic challenge (lowered SpO_2_ by ~32%) significantly increased MPN, f_R_, ʃPN and HR and decreased MABP in the Ctrl. These cardiorespiratory responses were profoundly affected by fentanyl. Specifically, fentanyl reduced the MPN, f_R_ and HR responses by 45%, 35% and 52% respectively and aggravated hypotension by 2.8-fold. P_ET_CO_2_ and T_B_ during hypoxia were maintained at the similar levels before and after fentanyl.

### IVb injection of fentanyl markedly attenuates the cardiorespiratory response to hypercapnia

**Figure 5:**
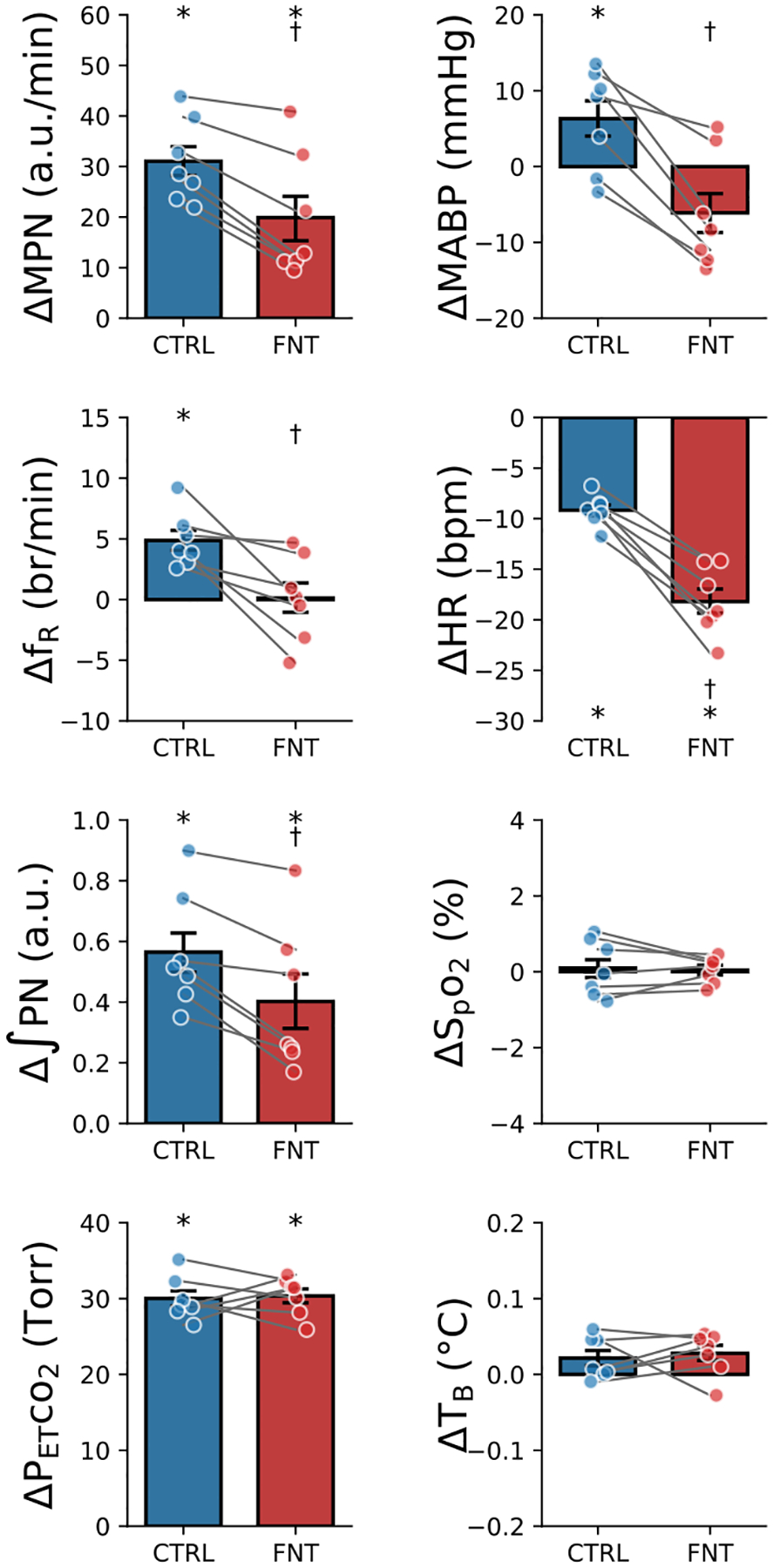
Cardiorespiratory responses to hypercapnia (10% CO_2_ for 3 min) before (CTRL) and after fentanyl (FNT) in anesthetized, vagotomized, paralyzed and ventilated rats. n = 7; mean ± SE; *, P < 0.05 compared to before hypercapnia; †, P < 0.05 compared to CTRL.

After recovery of the cardiorespiratory responses to hypoxia, hypercapnia (10% CO_2_ for 3 min) was applied before and after fentanyl. As displayed in Fig. 5, hypercapnia (elevating P_ET_CO_2_ by 30 torr) significantly augmented MPN, f_R_, ʃPN, and MABP and dropped HR in the Ctrl. The responses of MPN and ʃPN were attenuated and the f_R_ responses were eliminated by fentanyl. The hypercapnia-induced hypertension became hypotension, and bradycardia was aggravated after fentanyl injection. There was no difference in the levels of SpO_2_ and T_B_ during hypercapnia before and after fentanyl.

## Discussion

One of our major findings in the present study is the obvious depression of PN induced by IVb injection of fentanyl (30 μg/kg) in anesthetized, vagotomized, paralyzed and ventilated rats. The depression is composed by three phases: (I) the quick decline of PN, leading to an apnea with a-18 sec latency; (II) the extra long-lasting PN silence (apnea) for ~6 min; and (III) the sustained depression of MPN and ʃPN by 70% with the rapidly recovered f_R_. Moreover, fentanyl promotes hypertension and tachycardia (after a brief bradycardia) during and after the apnea. The difference in the persistent severe ʃPN depression and the rapid f_R_ recovery after apnea implies a more sustained impact of fentanyl on the respiratory pattern compared to the respiratory rhythm. Our previous study showed that IVb injection of fentanyl (15 or 25 μg/kg) triggered an immediate (with a-3 sec latency) and sudden apnea lasting for up to 35 sec in anesthetized, spontaneously breathing and vagal intact rats [4]. The apnea was followed by ventilatory depression (↓~75%) that was quickly and largely alleviated within 5 min postinjection, associated with slight hypertension and tachycardia. Clearly, the similarity of the reduction of MPN and V_E_ (70% and 75%) suggests that the decreased PN is the major contributor to the fentanyl-induced ventilatory depression.

Besides the similarity described above, compared to the anesthetized, spontaneously breathing and vagal intact rats, there are several distinct features of the apnea and the subsequent respiratory depression observed in this study: 1) the latency of the apnea is much longer (3 sec vs. 18 sec); 2) the apneic duration is extremely prolonged (35 sec vs. 6 min); and 3) the degree of the MPN recovery was much lower (75% vs. 30%) at the 10^th^ min postinjection. We believe that the absence of the immediate apnea in this study is the result of the vagotomy. IVb injection of fentanyl, morphine and dermorphin reportedly triggers an immediate apnea followed by subsequent respiratory depression. The immediate apnea, rather than the subsequent ventilatory depression, is eliminated by bilateral vagotomy, blockade of vagal C-fiber conduction and antagonism of vagal afferent ORs in rats [4, 42]. Moreover, the majority of vagal sensory C-neurons express MORs and these neurons are directly activated by fentanyl *in vitro* [4]. The delayed appearance of the apnea and the subsequent sustained respiratory depression noted in our vagotomized rats point to the central action of fentanyl. It is well-documented that opioids inhibit the respiratory-related neurons in the respiratory network located within the multiple pontomedullary regions, such as the pre-Botzinger Complex, post-inspiratory complex, nucleus tractus solitarius, medullary raphe and Kolliker–Fuse/Parabrachial nuclei [22, 43–45]. A recent study implicates direct fentanyl actions on phrenic motoneurons in mice [46]. Further study is required to determine the precise central mechanisms responsible for the fentanyl-induced three phases of phrenic nerve responses. In addition, the extra long-lasting nature of the apnea and the much slower recovery of subsequent PN depression in this study may be due to the lack of hypoxemia/hypercapnia that are able to accelerate respiratory rhythmic restoration [47, 48]. Collectively, our results provide the first evidence revealing the impacts of IVb injection of fentanyl on the inspiratory motor drive over time.

Another major finding in this study is the significant attenuation of PN response to hypoxia and hypercapnia by fentanyl. We found that hypoxia substantially augmented MPN, f_R_, and ʃPN associated with decrease in MABP and increase in HR and these responses were markedly blunted by fentanyl. On the other hand, hypercapnia elevated MPN, f_R_ and ʃPN associated with slight hypertension and bradycardia, and fentanyl markedly lowered the MPN and ʃPN responses and abolished the f_R_ response and exacerbated cardiovascular responses. Our results are in line with the majority of the previous studies showing the depressed HVR and HCVR by opioids (fentanyl) [24–27, 29–34], while there are a few reports indicating no effect of opioids on HVR [26, 27, 29]. This discrepancy results presumably from the different opioid doses and agonists (with the different potency) used. In support, a high, but not low, dose of fentanyl (iv) was reported to strikingly depress HVR in A/J mice [34]. HVR is triggered by stimulating the carotid body [49] and achieved by carotid body afferents’ synaptic projection to the nucleus tractus solitarius and further projections to the respiratory network [50–52]. The role of the carotid body in opioid-induced respiratory depression seems to be debatable. Carotid body afferent nerve activity was reported to be suppressed by enkephalins and morphine, but enhanced by naloxone during hypoxia, indicating an inhibitory influence of endogenous enkephalin-like peptide on carotid chemoreceptor [53]. In opposition to the inhibitory impact, a recent report pointed out that fentanyl stimulated carotid body afferents via acting on local κ-opioid receptors [54]. Thus, the investigation is warranted to clarify the role of the carotid body in the respiratory depression elicited by IVb injection of fentanyl. In fact, the nucleus tractus solitarius or medullary raphe are crucial for the carotid body-mediated chemoreflex [33, 55–58]. Activation of MORs in these regions has been demonstrated to abrogate or diminish HVR [33, 55]. With respect to HCVR, it is primarily mediated by central CO_2_/H^+^ chemo-sensitive neurons, such as those located in the retrotrapezoid nucleus, nucleus tractus solitarius and medullary raphe [59–61]. Activation of MORs in the medullary raphe region blunts HCVR in anesthetized rats and awake goats [31, 62]. On the other hand, a recent study indicated that fentanyl (500 μg/kg ip) brought about respiratory depression, but unexpectedly failed to inhibit the excitability of chemo-sensitive neurons of the retrotrapezoid nucleus in mice [63]. Certainly, the mechanisms by which fentanyl depresses the inspiratory motor drive during hypoxia and hypercapnia need to be further elucidated in future studies. Nevertheless, our results highlight the fentanyl-induced depression of inspiratory motor drive in response to hypoxia and hypercapnia.

In summary, in this study fentanyl depressed inspiratory motor drive and attenuated its response to hypoxia and hypercapnia in the animal preparation that lacks the vagal afferents and avoids the opioid-induced upper airway constriction, respiratory muscles rigidity, hypoxemia/hypercapnia and hypothermia. This, along with the fact that the blunted chemoreflexes are critical in the genesis of opioid-induced ventilatory depression [30, 35], suggest that the attenuated inspiratory motor drive during hypoxia and hypercapnia substantially contribute to the fentanyl-induced depression of HVR and HCVR. Opioid overdose-induced respiratory depression characterized by bradypnea and apnea as well as severe hypercapnia and hypoxemia, leading to fatal ventilatory arrest in therapeutic and illicit opioid users has been a critical medical challenge [64, 65]. Therefore, our findings are beneficial not only to better understand the pathophysiology of the opioid-induced respiratory depression but also target the interventions to alleviate the opioid-induced respiratory depression.

## Figures and Tables

**Table 1:** Time frames for data collections (mean ± SE)

Time-points	FNT (min)
T1	0.00 ± 0.00
T2	0.22 ± 0.07
T3	0.32 ± 0.10
T4	6.20 ± 0.52
T5	6.73 ± 0.50
T6	7.77 ± 0.56
T7	8.97 ± 0.71
T8	10.51 ± 0.7

## Abbreviations

ABP: arterial blood pressure
f_R_: frequency of ʃPN
HCVR: hypercapnic ventilatory response
HR: heart rate
HVR: hypoxic ventilatory response
IVb injection: intravenous bolus injection
MABP: mean ABP
MOR: Mu-Opioid receptor
MPN: minute phrenic activity
OR: Opioid receptor
P_ET_CO_2_: partial pressure of end-tidal carbon dioxide
PN: Phrenic nerve activity
ʃPN: the integrative phrenic nerve activity
SpO_2_: peripheral capillary oxygen saturation
T_B_: body temperature
